# Development of vegetable farming: a cause of the emergence of insecticide resistance in populations of *Anopheles gambiae *in urban areas of Benin

**DOI:** 10.1186/1475-2875-8-103

**Published:** 2009-05-14

**Authors:** Anges William M Yadouleton, Alex Asidi, Rousseau F Djouaka, James Braïma, Christian D Agossou, Martin C Akogbeto

**Affiliations:** 1 Centre de Recherche Entomologique de Cotonou, 06 BP 2604, Benin; 2 University of Abomey-calavi, Benin; 3 International Institute of Tropical Agriculture, 08 BP 0932 Cotonou, Benin

## Abstract

**Background:**

A fast development of urban agriculture has recently taken place in many areas in the Republic of Benin. This study aims to assess the rapid expansion of urban agriculture especially, its contribution to the emergence of insecticide resistance in populations of *Anopheles gambiae*.

**Methods:**

The protocol was based on the collection of sociological data by interviewing vegetable farmers regarding various agricultural practices and the types of pesticides used. Bioassay tests were performed to assess the susceptibility of malaria vectors to various agricultural insecticides and biochemical analysis were done to characterize molecular status of population of *An. gambiae*.

**Results:**

This research showed that:

(1) The rapid development of urban agriculture is related to unemployment observed in cities, rural exodus and the search for a balanced diet by urban populations;

(2) Urban agriculture increases the farmers' household income and their living standard;

(3) At a molecular level, PCR revealed the presence of three sub-species of *An. gambiae *(*An. gambiae s.s., Anopheles melas and Anopheles arabiensis*) and two molecular forms (M and S). The *kdr *west mutation recorded in samples from the three sites and more specifically on the M forms seems to be one of the major resistance mechanisms found in *An. gambiae *from agricultural areas. Insecticide susceptibility tests conducted during this research revealed a clear pattern of resistance to permethrin (76% mortality rate at Parakou; 23.5% at Porto-Novo and 17% at Cotonou).

**Conclusion:**

This study confirmed an increase activity of the vegetable farming in urban areas of Benin. This has led to the use of insecticide in an improper manner to control vegetable pests, thus exerting a huge selection pressure on mosquito larval population, which resulted to the emergence of insecticide resistance in malaria vectors.

## Background

Malaria is one the deadliest vector-borne disease in the world with 1.5 to 3 million deaths a year [1]. More than 90% of the deaths recorded occur in Africa affecting mainly low immune response individuals, such as children under five years of age and pregnant women [2,3]. In 1992, the WHO set up sustainable strategies against malaria, focused on the proper treatment of malaria cases and the use of preventive measures against malaria vectors. Indoor residual spraying (IRS) and long-lasting insecticidal nets (LLINs) remain the two preventive measures presently used against malaria vectors. Both methods have been very effective in controlling *Anopheles *mosquitoes [4-10]. However the emergence of *Anopheles gambiae *populations carrying the *kdr *gene has become a serious threat to the future effectiveness of these control measures [3]. N'Guessan *et al *[11] recently established a clear relationship between pyrethroid resistance caused by *kdr *and the failure of LLINs and IRS in experimental huts in south Benin.

In the last decade, the emergence of resistance in populations of *Anopheles *to common class of insecticides used in public health was reported from many African countries [7,8]. Resistance affects the major vectors of malaria, *An. gambiae s.l *[4] and *Anopheles funestus s.l *[5]. The resistance of pyrethroid insecticides in *An. gambiae *has been documented in several parts of Africa [8,9,12,13] and has prompted considerable research activity to investigate mechanisms of resistance and factors contributing to the emergence of insecticide resistance [11,14]. There is currently a growing agricultural activity within and around African cities. In Benin, the urban farming has spread in almost all the major cities all over the country. Akogbeto *et al *[14,15] reported that mosquito species, *An. gambiae *in particular, lay their eggs in breeding sites located around agricultural settings. These eggs undergo a selection pressure from agricultural pesticides, which leads to the emergence of resistant strains. There is clear evidence on the implication of agricultural breeding sites in the selection of resistance in the major malaria vectors.

Indeed, a fast development of urban agriculture has recently been recorded in most settings in the Republic of Benin. At all levels of the society, people are devoted themselves to it. The reason which underpins the phenomenon is the impoverishment of the soil far from the town due to its overuse, rural exodus, unemployment, improvement of living standards, and the dietary requirement of urban population to be met.

Being ideal environments for larval growth, it has been reported that vegetable farming uses a large variety of synthetic pesticides for pest control [16]. Some of these insecticides are registered for pest treatments in vegetable farms, whereas many are not [14-16]. During treatment, insecticide residues are washed away into the mosquito breeding sites thus exerting a selection pressure on larvae population [14,15]. This selection leads to the emergence of insecticide resistance in the population of *An. gambiae *breeding in these sites. The massive utilization of agricultural pesticides constitutes, therefore, a public health issue in tropical Africa [14,15].

This study was designed to assess the impact of the fast growing activities of vegetable farming on the resistance status of malaria vectors in three localities (Cotonou, Porto-Novo and Parakou) in Benin. The study focused on the investigation of agricultural practices in vegetable farming in urban areas, and their impact on the emergence of insecticide resistance in populations of *An. gambiae*.

## Methods

### Study sites

The study was conducted in the Republic of Benin, from July 2005 to February 2007 in three vegetable farms: Houeyiho in Cotonou, the economical capital of Benin, Acron in Porto-Novo, the political capital of Benin, and Azèrèkè near Parakou, in the northern part of the country.

#### The vegetable farm of Houeyiho, Cotonou

This farm is located at 6° 45'N and 2°31'E in the downtown of Cotonou in a highly populated quarter. It is a 14-hectare farm shared between five cooperatives, each led by a chosen cooperative president. Each cooperative approximately consists of 300 individuals making an estimated farmer population of not less than 2,000 persons.

#### The vegetable farm of Acron, Porto-Novo

Located in south-eastern Benin at 6° 30'N and 2°47'E at the outskirt of Porto-Novo, the vegetable farm of Acron is the oldest one in Benin. This site was established by missionaries in 1945. The farm consists of three hectares, nitially cultivated by 10 farmers. The activities of the farm have now grown and the size has widened from three to 20 hectares. The number of farmers has also increased to about 150 individuals.

#### The vegetable farm of Azèrèkè, Parakou

This farm is located at 9° 22'N and 2°40'E at the entrance of Parakou town, known as Azèrèkè site. The size of this vegetable plantation is 10 hectares. The farm is crossed by a canalization of rainfall from the main town. The vegetable farm of Azèrèkè is mostly cultivated by men of 35 to 50 years old and their children.

### Collection of data on the rapid spread of vegetable farms in Benin

To generate adequate information on the fast spread of vegetable farms in Benin, knowledge-attitude-practice (KAP) studies were organized in the study sites of Houeyiho, Acron and Azèrèkè. A total number of 150 farmers were interviewed at Houeyiho, 80 at Acron and 60 at Azèrèkè. Farmers were subjected to semi-structured questionnaires focused on the history of vegetable farms, the size of farms, the number of workers, their educational levels, the type of vegetable grown, the farming techniques, the pesticides utilization in the farm. Qualitative data were recorded from direct observations, in-depth interview and focus group discussion.

### Insecticide susceptibility test

To assess the impact of agricultural pesticides on the selection of resistance in malaria vectors, *Anopheles *larvae were collected from vegetable farms and reared to adults in the insectaria. Females mosquitoes aged 2–5 days old were subjected to susceptibility tests using insecticide-impregnated papers, as described by the WHO testing protocol [17]. Deltamethrin papers, impregnated at the diagnostic concentration of 0.05%, were used in this susceptibility assay. Results with this insecticide were compared with permethrin-impregnated paper (0.75%) and DDT-treated paper (4%). DDT and permethrin were both tested to detect the presence of cross-resistance between pyrethroids and organo-chlorine in *Anopheles *populations. Female *Anopheles *used in this bio-assay were exposed for one hour to insecticide-treated papers and were monitored at different time intervals (10', 15', 20', 30', 45', 60') to record "knock-down" times.

After 24-hour holding, delayed mortality was recorded. Following the WHO protocol, populations of *Anopheles *giving mortality rates below 95% after exposure to insecticide-impregnated papers were considered resistant. In this study, these criteria were slightly modified as follow:

- Mortality rates between 100-95%: the population was considered fully susceptible

- Mortality rates between 94-90%: the population was considered less susceptible

- Mortality rates below 90%: the population was considered resistant to the tested insecticides.

Based on the criteria mentioned above, data from insecticide susceptibility tests were used to characterize the susceptibility levels of *An. gambiae *populations in the three study sites. Dead and survived mosquitoes from this bioassay were separately kept in Carnoy solution at -20°C for further molecular characterization.

### Data analysis

Sociological information from focus group discussions, in-depth interviews and questionnaires conducted in studied communities were compiled and tabulated using Excel software and qualitative data were analysed by Text Base-Beta Software. Insecticide susceptibility test on the resistant strains from Houeyiho, Acron and Azèrèkè were compared and analysed using Stat-calc-Epi-info Software to get the status of resistance in the different sites investigated. A Fisher's exact test was performed to determine the differences between the three sites.

## Results

### Management of vegetable farms in Benin

At economical level, urban agriculture offers several advantages to farmers: quantitative and qualitative data from interviews and focus group discussion conducted in the three study sites revealed a major socio-economical impact of urban vegetable farming in households. Statistical studies conducted in vegetable farms of Benin for this research have shown that this activity produces each year more than 300 million FCFA to farmers [16,18]. This study has also shown that vegetable farming contributes to the development of husbandry by buying several end products and natural fertilizers like animal excreta. Vegetable farms consume each year more than 50 tones of natural fertilizers per hectare surface from husbandry, which represents one million franc CFA per hectare purchased to as fertilizers by aviculturist.

**Figure 1:**
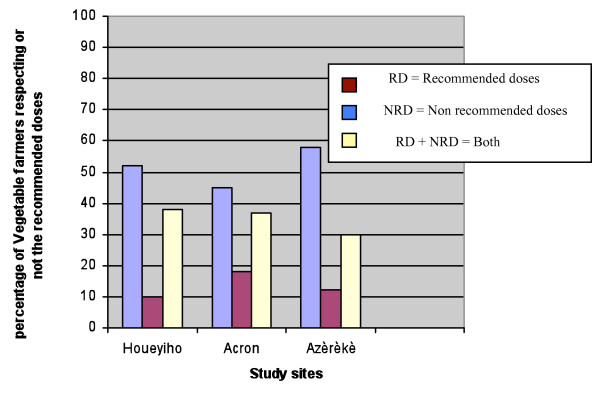
**Doses used by vegetable farmers at the three study sites**.

During investigation in the field, many pesticides were used but farmers declared that most of the insecticides were not registered for pest control in vegetable farms (Figure 1).

At social level, urban agriculture absorbs unemployed people by providing several job opportunities. In Cotonou, vegetable farms employ directly and permanently a total number of 3,600 workers, of which 600 are heads of groups and 3,000 are work members [16,18,19]. Subsequently, indirect beneficiaries, such as vegetable and pesticide sellers, also generate substantial incomes from vegetable farming. Series of training on agricultural practices are organized in these farms and are directed to unemployed youth.

### Susceptibility of vectors to agricultural insecticides

5,000 three-day old female *An. gambiae *were exposed to three insecticide-impregnated papers (permethrin at 0.75%; deltamethrin at 0.05% and DDT at 4%). Mortality in control tubes was less than 5%.

### Susceptibility of *An. gambiae *to pyrethroids

4,000 three- to five-day old female *An. gambiae *mosquitoes from Cotonou and Porto-Novo were exposed to permethrin. Mosquitoes tested with pyrethroid insecticides showed very low levels of mortalities (Table 1), which varied between 17% and 35.5% in Cotonou and 23.5% in the Porto-Novo site, respectively. The highest mortality rate (80.5%) was recorded during the rainy season with females from the vegetable farm of Houeyiho. A gradual lost of susceptibility to deltamethrin was observed with mortalities ranging from 86% to 90% in Cotonou and 91% in Porto-Novo.

**Table 1:** Pesticides commonly used in vegetable farms at the three study sites

Commercial name	Active ingredient	Family
Cyhalone	deltamethrin	pyrethroid

Decis	deltamethrin	pyrethroid

Kinikini	mixture of cyfluthrin+malathion	pyrethroid

Rugby (Orthene)	cadufos 10 g	pyrethroid

Cytoate 335EC (Cypercal D335)	mixture of cypermethrin 35 g + dimethoate 300 g/l	pyrethroid

Furadan	carbofuran	carbamate

Malathion	malathion 500 g/l	organochlorine

Topsin-M	methylthiophanate 70%	organophosphorus

Nurelle D35/300	mixture of cypermethrin 35 g/l + chlorpyriphos methyl 300 g/l	organophosphorus

Dursban B18/300	mixture of cyfluthrin 18 g/l + chlopyriphos ethyl 300 g/l	organophosphorus

Callisulfan 350EC	endosulfan 350 g/l	cyclodiene

Source: Benin Minsitry of Agriculture; 2007

A total number of 1,500 female *An. gambiae*, collected in vegetable farm of Parakou, was exposed to permethrin-impregnated papers. Mortality ranged from 76% to 80%. These mortality rates revealed the emergence of permethrin resistance in *An. gambiae *in this study site (Table 1). Concerning deltamethrin 0.05%, the mortality recorded ranged from 90.5 to 93.5%, suggesting the emergence of deltamethrin resistance in these populations.

### Cross-resistance to DDT and pyrethroids

Only specimens of *An. gambiae *collected at Cotonou and Porto-Novo sites were tested with DDT. In both localities, *Anopheles *were found resistant to permethrin and deltamethrin (Table 2). Low mortality rates were recorded with DDT (13.75% to 15.41%) (Table 3). This resistance observed with the DDT indicates a cross-resistance to the DDT and pyrethroids.

**Table 2:** Susceptibility of Anopheles gambiae s.s.* to permethrin and deltamethrin at the three study sites

	Permethrin (0.75%)		Deltamethrin (0.05%)	
	
	Number tested	% Mortality	Number tested	% Mortality
Houyiho	1500	18.5^a^	1500	97.5^a^
Acron	1500	22,5^a^	1500	98^a^
Azérèkè	1500	76.5^b^	1500	98.5^a^

NB. Numbers in the same column with the same superscript do not differ significantly by Fisher test (P > 0.05)

*Tested Anopheles were collected from the vegetable farms of Houeyiho, Acron and Azérèkè.

**Table 3:** Susceptibility of *Anopheles gambiae s.s. *to DDT at two study sites

	DDT			
	
	Number tested	Death after 24 h	Alive after 24 h	Mortality (%)
Houyiho	1200	165^a^	1045^a^	13.75^a^
Acron	1200	185^a^	1015^a^	15.41^a^

### Determination of molecular forms and identification of species characterizing vegetable farms

Table five shows that 95% of mosquitoes analysed by PCR in Cotonou were *An*. *gambiae s.s *and 5% were *An. melas*. In Porto-Novo, 92% were *An. gambiae *and 8% *An. melas*. In Parakou, 85% were *An. gambiae ss *and 15% *An. arabiensis*. The characterization of molecular forms of *An. gambiae *from the northern site of Parakou gave 35% of « M » forms and 65% of « S » forms. By contrast, in the southern Benin at Houeyiho and Acron sites only « M » forms was found (Table 4).

**Table 4:** PCR determination of mosquito forms and species collected from the three study sites.

	PCR (form)			PCR (Species)		
	Total tested	% S	% M	*% An. gambiae s.s.*	*% An. melas*	*% An. arabiensis*
Houyiho	200		200	198	2	---
Acron	200	----	200	192	8	---
Azêrekê	200	35	165	185	-----	15

### Frequency of kdr mutations (Leu-phe) on resistant populations of Anopheles from vegetable farms

The PCR analysis of mosquito samples from Houeyiho (Cotonou) and Akron (Porto-novo) revealed high frequencies of *kdr *mutation in *An. gambiae *populations: 88% in Houeyiho and 86.7% in Acron. This mutation was also found at 82.2% in samples of *An. gambiae *from Parakou (Table 5). However, analysis of *An. arabiensis *and *An. melas *collected during this study showed no *kdr *mutation. The *kdr *mutation is likely to be the main mechanism of resistance in *An. gambiae *in Benin.

**Table 5:** PCR characterization of *kdr *gene in *An. gambiae *collected from the three study sites

	PCR *kdr*							
	
	Survivors (Total tested per site: 200)				Dead (Total tested per site: 100)			
	
Study sites	RR	RS	SS	*Kdr *Frequency	RR	RS	SS	*Kdr *Frequency
Houeyiho	60	120	20	90^a^	7	20	73	27^a^
Acron	57	115	28	86^a^	12	23	65	35^a^
Azérèkè	45	98	57	71.5^a^	5	15	80	20^a^

NB. Numbers in the same column with the same superscript do not differ significantly by Fisher's test (P > 0.05)

## Discussion and conclusion

Information collected during the interviews with farmers and the direct observations made confirmed a fast growing activity of vegetable farming and their economical impact in Benin. Urban agriculture contributes to food security and balanced diets. It provides additional incomes to populations throughout the year.

A field study conducted in Benin revealed that vegetable farming yields about 300 million FCFA annually to farmers in Benin with 30 to 40% used for direct consumption in farmers daily diet [3,15,20]. There is a clear evidence that vegetable farming activities do absorb unemployment and eventually reduce hunger [16,19-21].

Based on its numerous advantages, vegetable farming is becoming a new activity involving the various social groups: men, women, and children, educated, non-educated and even civil servants. More than 3,000 individuals are employed by urban farming and this sector is mainly man-powered by young people between 20 and 40 years of age. The massive use of pesticides in vegetable farms was confirmed during interviews with farmers. Some of the pesticides recorded in vegetable farms were not registered, probably because of the liberalization of the pesticide sector and the elevated cost of registered pesticides (Table 1). Uncontrolled use of pesticides in Benin has resulted in the emergence of insecticide resistance in *An. gambiae *larvae breeding in vegetable farms. The emergence of pyrethroid resistance in *An. gambiae *has become a serious concern to the success of malaria control in the last decade [22,23]. This study showed that pyrethroid insecticides used in vegetable farms are similar to those used in public health against malaria vectors. Pyrethroids remain the only family of insecticides currently registered for the impregnation of bed-nets, the major control strategy against malaria vectors [6,24]. Among the pyrethroids, deltamethrin is definitely the most used in both public health and agriculture. Cyfluthrin is one of the pyrethroids used in combination with organophosphates in agriculture [14,15]. The *kinikini *(in local language), which is a combination of cyfluthrin and malathion, is widely used in vegetable farming and in public health as Solfac [14,15]. Studies on insecticide resistance have been currently on most malaria agenda in Africa because of its impact on impregnated bed-nets the major tool against malaria vectors [6,24]. The first case of pyrethroids resistance in *An. gambiae *has been reported in Africa since the 1993 [23,24]. Dieldrin and DDT resistance were reported in Burkina-Faso with populations of *An. gambiae *[23,24]. Pyrethroids resistance was reported in *An. gambiae *in Côte d'Ivoire [22,23] and later on many others cases of pyrethroid resistance in Anopheles vectors were detected in West [25], Central [24], Eastern [26] and Southern Africa [27].

In a recent study, a relatively high frequency of *kdr *mutations (*Leu-Phe*) was recorded in *An. gambiae *collected from cotton farms under massive insecticide treatments compared to farms with no pesticide utilization [22,23]. The *kdr *mutation is probably responsible for the emergence of resistance of *An. gambiae *to DDT and pyrethroids in West Africa. The hypothesis of the implication of the *kdr *mutation in the emergence of resistance has been confirmed in this research. In most localities where resistance was detected, the PCR analysis of samples revealed a high frequency of *Kdr *genes in localities of Cotonou and Porto-Novo. This study provides further evidence on the contribution of the overuse of insecticide in agriculture to the widespread emergence of insecticide resistance in *Anopheles *species. In addition to the *kdr *mutation, there are other existing factors, which seem to confer cross-resistance to pyrethroids and DDT, as described by Diabaté *et al *in the West African region [23].

The implication of metabolic mechanisms of resistance was not neglected in this study. Several studies are being currently conducted at CREC to determine the levels of acetylcholinesterase in *An. gambiae *after exposure to propoxur. In addition, elevated monooxygenase, esterases and glutathion-s-transferases have also been investigated. The increase number of vegetable farming in urban areas of Benin has been confirmed as a result of investigations made during this study from July 2005 to February 2007. This has led to the use of insecticide in improper manner to control vegetable pests, thus exerting a huge selection pressure on mosquito larval population leading to an emergence of mosquito resistance to insecticides.

More investigations need to be carried out in the future in order to better control the use of pesticides in vegetable farming within urban areas, especially in Benin where pyrethroid resistance has been widely reported in *An. gambiae*. These findings showed an increase emergence of resistance in *An. gambiae *populations in the vegetable farm breeding sites located not far from human dwellings. It is, therefore, important to set up insecticide management structures to prevent failure from malaria vector control measures using especially those pyrethroid insecticides.

## Competing interests

The authors declare that they have no competing interests.

## Authors' contributions

AWY contributed to design of the study and conceived the protocol, the data analysis and interpretation. MCA contributed in the study design, fully involved financially and in the implementation of this research, guided the study from conception to the manuscript finalization and the write up of the manuscript. JB contributed in the study design and in the implementation of this research. AA, RFD and CDA contributed to the design of the study and substantially helped in drafting the manuscript.
